# A Single Centre Experience of First “One Hundred Laparoscopic Liver Resections”

**DOI:** 10.1155/2014/930953

**Published:** 2014-02-11

**Authors:** S. Rehman, S. K. P. John, J. J. French, D. M. Manas, S. A. White

**Affiliations:** ^1^Department of Hepatobiliary and Transplantation Surgery, The Freeman Hospital, Newcastle upon Tyne NE7 7DN, UK; ^2^The Liver Research Group, The University of Newcastle, Leech Building, Framlington Place, Newcastle upon Tyne NE1 7RP, UK

## Abstract

*Background*. Laparoscopic liver resection (LLR) has emerged as an alternative procedure to open liver resection in selected patients. The purpose of this study was to describe our initial experience of 100 patients undergoing LLR. *Methods*. We analysed a prospectively maintained hepatobiliary database of 100 patients who underwent LLR between August 2007 and August 2012. Clinicopathological data were reviewed to evaluate surgical outcomes following LLR. *Results*. The median age was 64 and median BMI 27. Patients had a liver resection for either malignant lesions (*n* = 74) or benign lesions (*n* = 26). Commonly performed procedures were segmentectomy/metastectomy (*n* = 55), left lateral sectionectomy (LLS) (*n* = 26), or major hepatectomy (*n* = 19). Complete LLR was performed in 84 patients, 9 were converted to open and 7 hand-assisted. The most common indications were CRLM (*n* = 62), followed by hepatic adenoma (*n* = 9) or hepatocellular carcinoma (*n* = 7). The median operating time was 240 minutes and median blood loss was 250 mL. Major postoperative complications occurred in 9 patients. The median length of stay (LOS) was 5 days. One patient died within 30 days of liver resection. *Conclusions*. LLR is a safe and oncologically feasible procedure with comparable short-term perioperative outcomes to the open approach. However, further studies are necessary to determine long-term oncological outcomes.

## 1. Introduction 

Since the initial report in 1992 by Gagner et al. laparoscopic liver resection (LLR) has evolved for treating benign and malignant tumours of the liver in selected patients [1–5]. As the technical innovations in the field of laparoscopic surgery continue to evolve more and more minimally invasive liver surgery is being carried out in specialised centres [4–15]. A review by Nguyen et al. in 2009 reported outcomes following LLR of 2804 patients citing reduced intraoperative blood loss reduced postoperative morbidity and mortality, and shorter in-patient hospital stay [3], albeit with slightly longer operating times [2]. Similar results have been reported by other centres [9–14]. In general the benefits of LLR are less surgical trauma associated with less postoperative pain, reduced analgesic requirements, and an early return to normal daily activities [13–18]. However, such findings have never been proved by a randomised controlled trial, and starting one now would be difficult as LLR has already become an established procedure in most specialist laparoscopic HPB units [13–18].

LLR for benign lesions gained acceptance relatively early on [1–5]; however, enthusiasm for laparoscopic resection of malignant lesions developed rather slowly [1–3, 5, 7–13], the major obstacles being oncologic inadequacy [10, 11] and concerns over seeding of tumour cells at the time of surgery and perhaps an increased risk of tumour recurrence [10–13]. Moreover, in its infancy LLR carried with it concerns regarding an increased risk of intraoperative bleeding and air embolism [10]. However, recent advances in the surgical techniques and development of more laparoscopic devices have largely overcome these problems to a certain extent [15–21]. Nevertheless, its long-term oncological outcome and added benefit of improving quality of life are still yet to be proven [10–14]. Perhaps for most enthusiasts in the field minor LLRs have obvious advantages although major hepatectomy is still very controversial.

The aim of this study was to evaluate the clinical and oncological outcomes of our first 100 LLRs performed in a supraregional HPB and liver transplant unit in North East England.

## 2. Methods

We analysed a prospectively maintained HPB database for patients undergoing LLR between August 2007 and August 2012. The primary outcome measure was short-term surgical outcome. The secondary end points were midterm overall and disease-free survival. Three surgeons (D. M. Manas, J. J. French, and S. A. White) have performed LLR in our unit since 2007 and make up 10–15% of all liver resections. Before surgery, each patient was individually evaluated in our weekly multidisciplinary team (MDT) meeting with surgeons, pathologists, oncologists, gastroenterologists, and radiologists. All patients had an abdominal CT scan and liver-specific double-contrast magnetic resonance imaging (MRI).

Patients with good performance status, resectable liver disease, absence of extrahepatic disease, and sufficient functional parenchyma were considered suitable for LLR. Patients with tumours within 1 cm of the portal vein bifurcation, the inferior vena cava, or hepatic vein confluence and tumours involving the common hepatic duct were found unsuitable for LLR. Moreover, large tumours (>10 cm) and the need for a portal lymphadenectomy were also considered to be contra-indications for a LLR in our early series.

Patient demographics, indications, type of liver resection, intraoperative blood loss, duration of surgery (time from start of skin incision to the end of wound closure), conversion to open or hand-assisted, length of intensive care unit (ICU) stay, postoperative length of stay (LOS), postoperative complications, and mortality (within 30 days from surgery) were evaluated. The histological reports were all reviewed to assess resection margin status.

The extent of hepatic resection was recorded according to the Brisbane 2000 terminology of liver anatomy and resections [9]. Operative results and postoperative variables were analysed for minor resections and major hepatectomies (e.g., removal of three or more segments, right hepatectomy, or left hepatectomy) where appropriate [3]. Postoperative complications were classified as per the Dindo et al. [16] classification. Margin status was defined as *R*_0_ when microscopically negative for tumour or *R*_1_ for microscopically positive for tumour existing within 1 mm of the margin.

## 3. Surgical Technique

### 3.1. Patient Positioning

For resections of the left lateral segment and tumours in the anterior segments, for example, IVb, V, and VI, we preferred a supine position with split legs with the surgeon standing between the legs and assistants on either side. Five ports (ENDO PATH Xcel, Ethicon Endo-Surgery, LLC, USA) including three 12 mm ports are positioned, one supraumbilical port, two in the right and left midclavicular line, and two 5 mm ports in the right and left anterior axillary line as described previously [17], but there has been a decrease in the number of ports with more experience.

### 3.2. Pringle's Manoeuvre

We always perform a staging laparoscopy to rule out extrahepatic disease at the time of the LLR. As part of the protocol a laparoscopic ultrasound (7.5 MHz, Aloka Co. Ltd., Tokyo, Japan) is performed to define the vascular anatomy and to confirm the location of metastases. Although various techniques for retracting the liver have been used in our series, the authors prefer to divide the falciform ligament and then place an Endoloop (Autosuture, Tyco Healthcare Ltd.) around the free edge of the ligamentum teres. This can be retracted superiorly by bringing the suture through the anterior abdominal wall using an Endo Close (Autosuture, Tyco Healthcare UK Ltd.) device. The suture is then held in a haemostat thus holding the ligament against the anterior abdominal wall or laterally. The gall bladder can also be used for retraction but some patients may have already had this removed.

Once the liver has been retracted and the hepato-duodenal ligament has been lifted a tape can then be placed, acting as a tourniquet around the hepatoduodenal ligament using a “Gold finger” (Gold finger, blunt dissector, Ethicon Endo surgery, Johnson & Johnson, USA) as previously described [17]. A nylon tape is passed through the snare in the tip of the Gold finger (Ethicon Endo-Surgery, Johnson & Johnson, USA). The Gold finger can be safely introduced through a 10 mm working port in the right upper quadrant due to its blunt and atraumatic tip. The Gold finger is then advanced around the porta hepatis until the tip of the nylon tape can be visualised on the left side of the hepatoduodenal ligament. The tape is then grasped through the port placed in the left upper quadrant in the midclavicular line. The two ends are positioned through the port onto the anterior abdominal wall and placed through a “snugger” using tubing (Suction tubing 10 cm, 7 mm, Pennine Healthcare Ltd., UK). The port is removed and then replaced with the tape lying adjacent on the outside of the port. With increasing experience this step has sometimes not been required at all.

### 3.3. Hilar Dissection and Parenchymal Transection

All major structures at the hilum are divided extrahepatically except the hepatic bile duct which is divided within the liver parenchyma using a suitable stapling device. Vascular staplers with roticulators are used to manage major pedicles and vessels. For right hepatectomy the right hepatic artery (RHA) and the right portal vein (RPV) are approached either anteriorly or laterally, usually posterior to the bile duct using locking Weck Clips. The Glissonian approach as described by Launois and Jamieson [21] was never used. Parenchymal transection was performed using either a combination of the cavitational ultrasonic aspirator (CUSA) or bipolar sealing device Tissue Link, the Harmonic Scalpel ultrasonic activated shears (Harmonic ACE) or Ligasure device. Tissue sealants such as Tisseel or Evicel were applied to the cut surface of the liver to further control bleeding from the parenchymal transection margin. The specimen is retrieved in an Endo-2 catch bag through a Pfannenstiel incision in most cases or through the previous midline incision if the indication was colorectal liver metastases (CRLM).

### 3.4. Followup

After initial followup between 4 to 6 weeks, all patients were regularly reviewed thereafter at 3, 6, 12, 18, and 24 months and yearly thereafter for the first 5 years for patients with malignant tumours. Patients with benign liver tumours were followed appropriately depending on their underlying pathological condition. Survival status was determined by review of the patients' medical records and defined as the time interval from the date of initial operation to the date of last clinical encounter or date of death if known.

### 3.5. Statistical Analysis

All results are expressed as median and ranges. The Mann-Whitney *U* test was applied to compare nonparametric data and the chi-squared test or Fisher's exact test were applied for analysis of categorical variables. Overall and disease-free survival was analysed by the Kaplan-Meier method and their significance was assessed using the log-rank test. The level of statistical significance was set at *P* < 0.05.

## 4. Results

74 patients had LLR for malignant disease, whereas 26 patients had resections for benign disease. There were 52 female and 48 male patients. The median age of all patients was 64 years (range 23–84 years) and median BMI was 27 (range 16–40) (Table 1).

Indications for surgery in the malignancy group were CRLM (*n* = 62), hepatocellular carcinoma (HCC *n* = 7), intrahepatic cholangiocarcinoma (*n* = 3), lymphoma (*n* = 1), and metastases from breast cancer (*n* = 1). Among the benign conditions the most common indications were adenoma (*n* = 9), biliary/liver cyst (*n* = 6), haemangioma (*n* = 5) (4 of these patients had a primary colorectal tumour and were suspected CRLM; however, histology of the resected liver revealed haemangioma, whereas one other patient had underlying ovarian primary tumour and was found to have indeterminate liver lesion, liver resection revealing a haemangioma), focal nodular hyperplasia (FNH *n* = 4), and angiomyolipoma (*n* = 2) (Table 2). The patient with lymphoma had a previous primary colorectal tumour that was thought to be a solitary secondary metastasis.

Major hepatectomies were performed in 19 patients (19%) and included 7 right hemihepatectomies, 6 left hemihepatectomies, 2 extended left hemihepatectomies, and 4 trisegmentectomies. Out of the 19 major hepatectomies, five patients were converted to an open procedure and two patients had hand-assisted liver resection (HALR) (Tables 5 and 7). In our series complete laparoscopic liver resection was performed in 84 patients (84/100), 9 were converted to an open procedure (Tables 3, 5, and 7) and 7 patients had a hand-assisted surgical resection (HALR) (Tables 1 and 7). For the hand-assisted technique a minilaparotomy in the right upper quadrant or insertion of a handport was used for completion of the parenchymal transection (Table 4).

The median overall operative time was 240 minutes (range 45–540 minutes), with a median blood loss of 250 mL (range 30–1200 mL). The median length of stay was 5 days (range 1–23 days) with a median of 1 day in either the ITU or HDU (1–8 days) (Table 6). A blood transfusion was required in only 11 patients. The median postoperative opiate requirement was 40 mg (range 30–100 mg). Moreover, in patients undergoing a major hepatectomy, the median operating time was 302 minutes (252–540), the median blood loss was 481 mLs (range 282–689), and median length of stay was 8 days (range 3–23 days) (Table 7). For patients undergoing LLS the median operative time was 195 minutes (range 45–285 minutes), median blood loss was 175 mLs (range 100–450 mL), median duration of analgesia requirement was 34 hours (range 8–62 hours), and median duration of hospital stay was 3 days (range 1–14 days) (Table 6).

Significant postoperative complications (Clavien-Dindo III/IV) occurred in 9 cases (9%) (Table 8). A bile leak requiring conservative management or stent placement was the most common complication (*n* = 3). A laparoscopic wash out was needed in 2 patients, one for an intra-abdominal haematoma and a second for an infected fluid collection, whilst another patient having a right-sided subphrenic fluid collection underwent an insertion of percutaneous radiological drainage. Two patients developed chest infections postoperatively requiring intravenous antibiotics, oxygen therapy, and chest physiotherapy; however, one of them deteriorated further and was shifted to the high dependency unit. One patient was readmitted 4 weeks after liver resection with signs and symptoms of small bowel obstruction, a CT scan confirmed findings of small bowel obstruction and thickening around a previous anastomosis. However, at laparotomy recurrence of the primary tumour was found at the previous anastomosis not detectable on previous imaging; the tumour was resected completely (Table 8). In-patient mortality was 1%. This patient developed a large pulmonary embolism and died two days after the surgery. The patient was on prophylactic Tinzaparin while on the ward.

**Figure 1:**
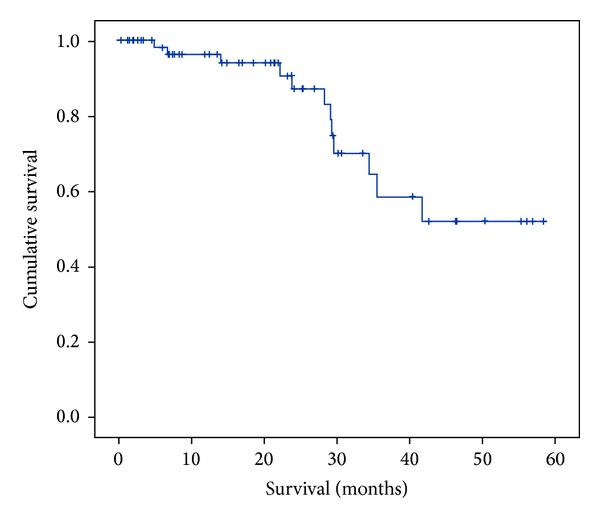
Overall survival for CRLM after LLR.

In a carefully selected subset of patients undergoing LLR for malignancy, complete surgical resection (*R*_0_) was achieved in 89% (*n* = 62, *R*_1_ resection = 7) CRLM patients and 72% (*n* = 7, *R*_1_ resection = 2) for patients with HCC. The median follow-up period was 14 months for CRLM (0.2–50 months) and HCC (11–40 months), respectively. The median recurrence-free survival was 18 months (range 8–28 months) in CRLM and 32 months (range 8–51 months) in HCC patients. Overall 3-year survival for CRLM patients was 78% with a median survival of 47 months (38–56 months) (Figure 1). A total of 10 patients died in the follow-up period, 8 due to progression of underlying disease. One patient died due to a cardiovascular event (3 years after liver resection) and one patient developed sepsis from a pneumonia and died from multiorgan failure 4 months after the liver resection.

## 5. Discussion 

LLR has been established as a favourable alternative to an open procedure to treat both benign and malignant diseases of the liver [1–6] and hepatobiliary surgeons around the world are increasingly performing LLR with greater confidence [7–13]. However, concerns still remain regarding parenchymal transection methods, controlling bleeding, bile leaks, and incomplete resection [3, 14–18]. This study suggests that LLR is a feasible and safe procedure in selected patients. Patient selection for LLR is still an issue and careful consideration must be given to the indications for LLR, particularly the position of the tumour, whether it is multifocal, and whether the patient can withstand a prolonged pneumoperitoneum. In our department each individual patient was discussed in a multidisciplinary team meeting (MDT) comprising hepatobiliary surgeons, a radiologist, a pathologist, and gastroenterologists; the decision regarding LLR was mutually agreed. Generally patients with a large tumour (>10 cm) and those involving major vasculature, for example, inferior vena cava, or with invasion of other adjacent organs were found unsuitable for LLR but not exclusively.

LLS and wedge resections were the most frequently performed procedures. The Louisville Statement, 2008, has recommended LLS as a standard technique for resection of left-sided liver tumours [4] but in our experience we would advise not taking on these types of resections early on when tumours are close to the MHV/LHV confluence. In our series the majority of patients had either nonanatomical resections of segments (II, III, IVb, V, and VI) or LLS. Major hepatectomies were performed in 19 cases (19%). Major hepatectomy (right or left hepatectomy or trisegmentectomy) is a much more complex and technically demanding procedure [17, 18] and can be associated with increased intraoperative bleeding resulting in reduced exposure of the transection plane and potentially the tumour margins [17–20]. Abu Hilal et al. in their study of 133 patients undergoing LLR for liver malignancies presented data of 42 major hepatectomies [8]. Their results revealed major hepatectomies resulting in increased blood loss, increased operating time, and increased conversion rate [8]. Our results are similar to their findings. However, we maintained a low threshold for conversion to an open procedure if the tumour size was large and difficult to manipulate and mobilize or hemostasis was a major concern; sometimes these issues were overcome by using a hand-assisted procedure.

Our results have shown that the overall median operating time was 240 minutes (range 45–540 min) which is consistent with most previous reports [8–21]. Some individual reports comparing LLR to an open approach have reported shorter operating times for open hepatic resection [21]. However, a recent systematic review has found no significant difference between laparoscopic and open liver resection with regards to operating time [15]. Indeed with regards to LLS this is now quicker than an open approach in our centre.

One added benefit of LLR is less intraoperative blood loss and reduced blood transfusion requirements [2–10]. Our results revealed median intraoperative blood loss of 250 mL (range 30–1200 mL), similar to others [6, 12, 13, 20–28]. In our experience only 11 patients required a blood transfusion which is also similar to others [20–28]. We believe laparoscopic surgery provides better visualization of deep vascular structures with more precise and accurate surgery for tumours located in the left lateral and anterior segments. We used a combination of various devices for parenchymal transection to avoid excessive bleeding. These included a Cavitron ultrasonic surgical aspirator (CUSA), Ligasure and the Tissue link together facilitating excellent haemostasis and clear anatomy.

LLR causes less tissue trauma consequently reducing postoperative morbidity [3–9]. In our series only 9 patients developed clinically significant complications. Our results corroborate those of previous studies [7–11]. Another advantage of LLR is minimal scarring and potentially fewer adhesions thereby increasing the feasibility of a repeat liver resection [11]. Recent reports suggest no significant survival difference between primary and repeat liver resection [13–17].

Proponents of laparoscopic surgery claim shorter in- hospital stays for patients undergoing LLR [11–19]. Our results revealed an in-patient stay of 5 days (range 1–23 days). These results support the findings of previous reports in the literature [3–9]. Using our learning curve and enhanced recovery techniques LLS patients can be discharged home the next day or in most cases on the 2nd or 3rd postoperative day. Shorter hospital stay helps reduce cost for organisations during difficult economic circumstances [12]. However, overall cost effectiveness of the procedure is also dependent on theatre time and the cost of instruments, which critics believe is much higher in laparoscopic surgery [20]. Recent reports in the literature suggest equivalent cost for open and LLR [9, 13]. In addition, introduction of enhanced recovery programmes in hepatobiliary surgery may further increase the cost-effectiveness of LLR. However, only future studies looking specifically at economic evaluation of laparoscopic and open liver resection during the era of enhanced recovery programmes would be able to answer these questions.

Oncologic adequacy of LLR has frequently been reported in the literature [5–9, 13–17] and there is a trend amongst most hepatobiliary surgeons that LLR is a safe and an oncologically feasible procedure in carefully selected patients [3–14]. Our results have shown that of 74 patients undergoing LLR for malignant lesions complete surgical resection was achieved in 89% of patients with CRLM and 72% in patients with HCC. These results coincide well with previous reports [19–27]. Without any treatment the median survival of patients with CRLM is 6 to 9 months [26] but now long-term survival after liver resection has been reported to be up to 60% in some studies [2, 3, 7, 13–25]. Our results revealed a median disease-free survival of 18 months (range 8–28 months) in those with CRLM and 32 months (range 8–51 months) in those with HCC although numbers are far too small in the latter case to make any useful conclusion. Moreover, overall 3-year survival following laparoscopic liver resection for CRLM was 78%. Abu Hilal et al. in their study of 133 patients undergoing LLR for various hepatic malignancies reported a 78% overall 2-year survival and 64% disease-free survival for CRLM patients [8]. They have also reported a 77% overall 2-year survival in HCC patients [6]. Our survival data are comparable with various other series in the literature demonstrating feasible medium term survival following liver resection for malignant lesions [13–31].

Important limitations to our study are the relatively small sample size, yet it is still one of the largest series reported in Europe. Nonetheless, our results coincide with the majority of previously reported series [9–19]. Furthermore, in the absence of a randomised controlled trial, case series will continue to provide further evidence to our existing knowledge regarding LLR. Major obstacles to conducting a RCT would be patient selection and randomising to an “open resection” as LLR has already become the gold standard in some specialist centres.

## Figures and Tables

**Table 1:** Patients characteristics and surgical outcome.

Variable	Frequency
Age (median in years, range)	64 (22–84)
Sex (female : male)	52 : 48
BMI (median)	27 (16–40)
ASA grade (median)	2
Laparoscopic	84
HALR	7
Converted	9

**Table 2:** Histological results in LLR *n* = 100.

Malignant tumour	*n* = 74
Colorectal liver metastases	62
Hepatocellular carcinoma	7
Cholangiocarcinoma	3
Metastases from breast cancer	1
Lymphoma	1
Benign tumour	*n* = 26
Adenoma	9
Biliary/liver cyst	6
Haemangioma	5
Focal nodular hyperplasia (FNH)	4
Angiomyolipoma	2

**Table 3:** Type of liver resection.

Types of liver resection *n* = 100	
Anatomical liver resection (major)	19
Right hemihepatectomy	7
Left hemihepatectomy	6
Extended L hemihepatectomy	2
Trisegmentectomy	4
Nonanatomical liver resection	55
Left lateral sectionectomy (LLS)	26

**Table 4:** Location of tumours.

Segmental position of liver tumour	Frequency (*n* = 81)
II	3
LLS (II, III)	16
III	7
IV, IVB	8
IV, V	6
V	9
V, VI	7
VI	8
VI, VII	12
VII	3
VIII	2

Major hepatectomy (*n* = 19).

**Table 5:** Reasons for conversion to an open procedure.

Reason	Frequency (*n* = 9)
Tumour in close proximity to large vessel and concern over margin status	4
Difficult/prolonged hilar dissection	2
Unable to locate the tumour	1
Large bulky tumour/bleeding	2

**Table 6:** Perioperative outcome following LLR.

Variable	Frequency
Size of tumour (mm)	35 (2–80)
Operation time (min)	240 (45–540)
Blood loss (mLs)	250 (30–1200)
Hospital stay (days)	5 (1–22)
Morbidity rate (%)	9 (9)
30-day mortality rate	1 (1%)

Median values.

**Table 7:** Major hepatectomy.

Variable	Major hepatectomy (*n* = 19)
Operation time (mins)	302 (252-353)
Blood loss (mls)	481 (282–689)
Hospital stay (days)	8 (3–23)
Open conversion	5
HALR	2

Median (range values).

**Table 8:** Postoperative complications.

Complications	Frequency	Management
Bile leak	3	Conservative management (*n* = 2) Biliary stent placement (*n* = 1)
Intra-abdominal collection/hematoma	3	2-laparoscopic washout (*n* = 2) Percutaneous radiological drainage
Small bowel obstruction	1	Required laparotomy, small bowel resection at day 27
Chest infections	2	Treated with antibiotic (*n* = 2) One required ITU admission
